# A Quality Control Mechanism of Splice Site Selection Abrogated under Stress and in Cancer

**DOI:** 10.3390/cancers14071750

**Published:** 2022-03-30

**Authors:** Maram Arafat, Ruth Sperling

**Affiliations:** Department of Genetics, The Hebrew University of Jerusalem, Jerusalem 91904, Israel; maram.arafat@mail.huji.ac.il

**Keywords:** pre-mRNA splicing, alternative splicing, latent splicing, aberrant splicing, endogenous spliceosome, breast cancer, glioma

## Abstract

**Simple Summary:**

Splicing and alternative splicing play a major role in regulating gene expression, and mis-regulation of splicing can lead to several diseases, including cancer. The aim of this review is to summarize the current knowledge of a quality control mechanism of splice site selection termed Suppression of Splicing (SOS), proposed to protect cells from splicing at the numerous intronic unused 5′ splice sites, and emphasize its relevance to cancer. This relevance stems from the finding that SOS is abrogated under stress and in cancer resulting in the expression of thousands of aberrant nonsense mRNAs that may be toxic to cells. These findings highlight the unexplored potential of such aberrant isoforms as novel targets for cancer diagnosis and therapies.

**Abstract:**

Latent 5’ splice sites, highly abundant in human introns, are not normally used. This led to the proposal of a quality control mechanism, Suppression of Splicing (SOS), which protects cells from splicing at the numerous intronic latent sites, and whose activation can generate nonsense mRNAs. SOS was shown to be independent of Nonsense-Mediated mRNA Decay (NMD). Efforts to decipher the SOS mechanism revealed a pivotal role for initiator-tRNA, independent of protein translation. Recently, nucleolin (a multifunctional protein) was found to directly and specifically bind the initiator-tRNA in the nucleus and was shown to be a protein component of SOS, enabling an updated model of the SOS mechanism. Importantly, SOS is abrogated under stress and in cancer (e.g., in breast cancer cells and gliomas), generating thousands of nonsense mRNAs due to activation of latent splicing. The resulting affected human genes cover a variety of functional groups, including genes involved in cell proliferation and differentiation. Furthermore, in oligodendroglioma, the extent of activation of latent splicing increases with the severity of the cancer. Interesting examples are genes expressing aberrant nonsense mRNAs in both breast cancer and glioma, due to latent splicing activation. These findings highlight the unexplored potential of such aberrant isoforms as novel targets for cancer diagnosis and therapies.

## 1. Introduction

Following transcription by RNA Polymerase II (Pol II), eukaryotic pre-mRNAs undergo a series of processing events, including 5′ and 3′ end processing, editing, and splicing, before exiting the nucleus. Pre-mRNA splicing occurs in the context of a large nuclear ribonucleoprotein (RNP) complex, termed the spliceosome.

Splicing and alternative splicing (AS) must be tightly regulated, as they have profound effects on gene expression. Various cis-regulatory elements control the fidelity and efficiency of splicing. These include the 5′ and 3′ splice sites (SSs), splicing enhancers, splicing silencers, branch points, and polypyrimidine tracts. These cis-regulatory elements are recognized, in turn, by small nuclear RNP (snRNP) and non-snRNP trans-acting factors, such as the uridine-rich snRNPs (U1, U2, U4, U5, and U6 snRNPs), the serine/arginine-rich splicing factors, and the heterogenous nuclear ribonucleoproteins (hnRNP) [1,2].

Aberrant splicing has been implicated in cancer and other diseases, and even small errors can be deleterious [3,4,5]. In view of the complexity of the splicing events and the requirement for accuracy, quality control of the mRNA products is critical. Aberrant transcripts that contain premature termination codons (PTCs) can be toxic, as they are translated into truncated proteins, which may be non-functional or, alternatively, may exert dominant negative effects. Thus, RNA surveillance mechanisms have evolved, which detect and degrade aberrant mRNAs, or prevent their production. A number of mechanisms have been demonstrated to be involved in RNA quality control processes that downregulate or eliminate the introduction of PTCs into mRNAs: I. Nonsense mediated mRNA decay (NMD) pathway, which can be considered as a post-splicing pathway, is the most studied RNA surveillance mechanism. It targets mRNAs undergoing premature translation termination for rapid degradation. Activation of NMD requires functional Upf factors, with Upf1 as the principal regulator of NMD. NMD was initially viewed as a mechanism for degrading defective mRNAs harboring PTCs, however, subsequently, it was also shown to target many mRNAs encoding functional full-length proteins [6,7,8]. Two additional models of a nuclear mechanism through which PTCs are eliminated by AS were proposed. II. Nonsense-mediated altered splicing (NAS), in which the PTC is eliminated through AS [9,10,11]. To date, the molecular mechanism of NAS is poorly understood, although two classes of NAS have been proposed: a splice motif disruption and a scanning mechanism; experimental evidence support both classes [9,10,11]. III. Suppression of Splicing (SOS), a third mechanism which occurs in the nucleus, is a stop codon-mediated suppression of splicing, in which splicing events at alternative latent (silent) intronic 5’ SSs are suppressed, thus preventing the inclusion of abundant intronic stop codons into the spliced mRNA [12]. A question pertaining to both NAS (scanning model) and SOS is how a protein reading frame can be recognized in the cell nucleus prior to splicing, as this requires the establishment of an open reading frame (ORF), yet this recognition does not necessarily depend on protein translation.

Herein, we will focus on the SOS mechanism of splicing regulation. This mechanism was proposed to suppress splicing at intronic latent 5′ SSs (LSSs) to avoid the introduction of intronic PTCs into the mRNA. It should be noted that SOS relates to naturally abundant intronic LSSs that could lead to production of thousands of nonsense mRNAs when SOS is abrogated (e.g., in stress and in cancer). Thus, SOS has an impact on the cell transcriptome, affecting a large variety of cellular functions. These findings highlight the importance of deciphering the mechanism underlying the lack of latent splicing in normal cells, with a view of understanding the reasons it is abrogated under stress and in cancer, resulting in splicing that generates thousands of unexplored nonsense mRNAs.

## 2. Alternative Splicing (AS) Is a Key Regulator of Human Gene Expression

Pre-mRNA splicing is an essential mechanism that controls the inclusion of exons and removal of introns in mature mRNA. Splicing and AS play major roles in regulating gene expression. More than 94% of human genes are estimated to undergo AS [5,13,14,15,16,17], which is considered to be a major source of the diversity of the human proteome. AS has been shown to control almost every aspect of protein function, such as protein localization, enzymatic activity, protein stability, and ligand interaction [4], and thus plays a crucial role in generating tissue and cell-type specific gene expression. Therefore, changes in AS and mis-regulation of AS factors are involved in numerous human diseases including cancer [3,5,18,19,20,21]. Through studies in genetics, molecular biology, and high-resolution cryo-EM, the molecular mechanism of splice site recognition and splicing of pre-mRNAs harboring a single intron is well understood [1,2,22,23,24,25,26,27]. However, it is still not clear how alternative SSs are recognized and selected in pre-mRNAs that contain multiple introns, and currently it is impossible to predict tissue-specific splicing programs.

Approximately 2500 transcription factors are known to regulate ~22,000 genes [28], yet only around 70 sequence-specific splicing regulators have been described [29]. Since both AS and promoter recognition are regulated through the combinatorial control of protein factors binding to either DNA or RNA, this scientific challenge prevents a full understanding of human gene regulation.

## 3. The Endogenous Spliceosome

Nuclear processing of Pol II transcripts, including splicing and AS, takes place in the endogenous spliceosome—the supraspliceosome. This is an enormous (21 MDa) highly dynamic structure comprising four active native spliceosomes joined together by the pre-mRNA [12,30]. The supraspliceosome is an autonomous macromolecular machine, where all nuclear pre-mRNAs, regardless of their length or number of introns, are individually assembled and processed, in a carefully controlled, coordinated fashion. The tetrameric structure of the supraspliceosomes is suitable to coordinate multiple processing events of the pre-mRNA. Accordingly, in previous studies it was shown that AS is regulated in supraspliceosomes [12,30].

## 4. A Quality Control Mechanism of Splicing Regulation

**Figure 1:**
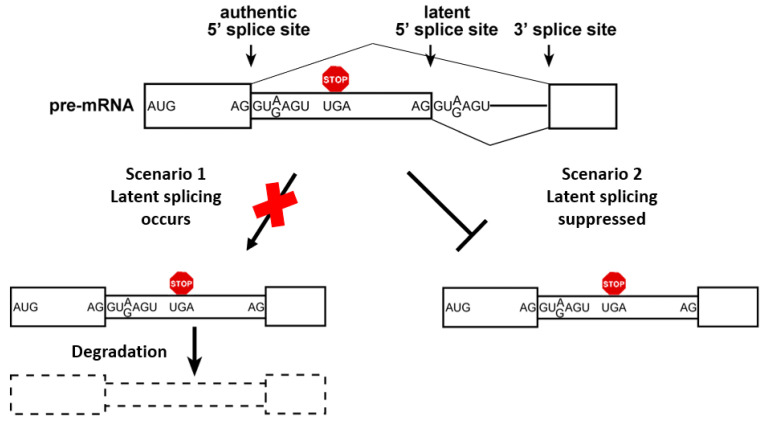
Suppression of splicing (SOS). Two scenarios for lack of latent splicing under normal growth conditions: scheme depicting the two scenarios that can account for lack of latent splicing under normal growth conditions, despite the abundance of LSS sequences in introns. As discussed, our studies have ruled out the first scenario [35,36,39,40]. Boxes, exons; narrow boxes, latent exons; lines, introns; red octagon, stop codon. Adapted from ref. [12].

Recognition and selection of the 5′ SS consensus sequence is a key step in pre-mRNA splicing [14]. Intriguingly, such potential sequences are abundant within human introns [31], but are not used under normal growth conditions, thus termed LSSs. Their use or activation could potentially add intronic sequences containing PTCs into most (98%) alternatively spliced isoforms [31], generating non-functional mRNAs that would be subjected to NMD in the cytoplasm [32,33,34]. Importantly, LSSs are activated under stress and in cancer [31,35,36], resulting in thousands of activated, aberrant gene transcripts [31]. Clearly, maintaining the fidelity of normal splicing is important and tightly controlled, but this can be derailed under certain conditions. Two scenarios might account for the lack of observed splicing at LSSs under normal growth conditions: (i) splicing at LSSs does occur, but an RNA surveillance mechanism, such as NMD [8,37,38], rapidly and efficiently degrades the nonsense mRNAs; (ii) a novel quality control mechanism suppresses splicing at LSSs that are preceded by at least one stop codon in-frame with the upstream exon (Figure 1). Experiments in our lab have ruled out the first scenario of NMD [35,36,39], as well as degradation of latent mRNAs by a yet unknown RNA degradation mechanism [40], while fitting the second scenario of latent splicing suppression. These discoveries led us to suggest a quality control mechanism of pre-mRNA splicing, which we named SOS. This mechanism, which differentiates between normal and LSS, requires recognition of an ORF that enables splicing only at normal 5′SSs, thus avoiding the generation of nonsense RNA transcripts [12,31]. Our results show that SOS is an evolutionarily conserved mechanism, likely shared by most eukaryotes [12,36]. Support for a nuclear surveillance mechanism that operates independently of NMD, recognizes PTC-harboring pre-mRNAs in the nucleus and suppresses splicing to prevent the production of such transcripts has been observed in a number of studies [41,42,43,44]. However, the mechanism of the SOS quality control remains nebulous.

## 5. Elements of the SOS Mechanism

### 5.1. SOS Requires Recognition of the Reading Frame in the Nucleus, Independent of Translation

**Figure 2:**
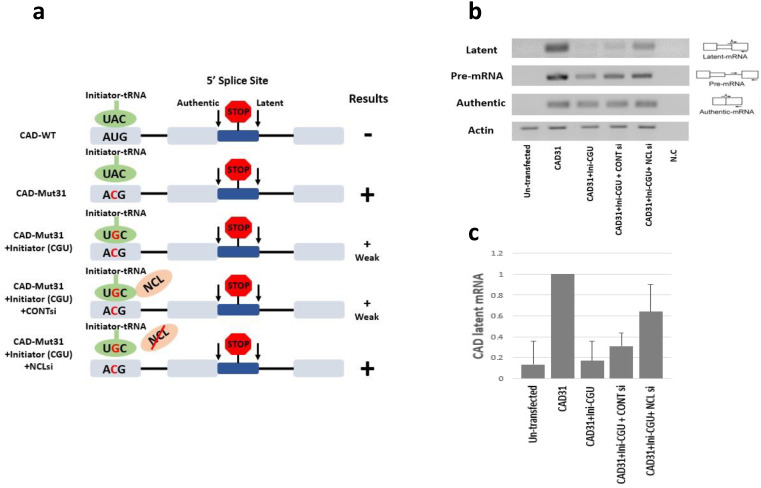
A potential role for NCL in SOS. The recovery of SOS by ini-tRNA complementation is NCL dependent. (**a**) Hypothesis and experimental design. Assuming that ini-tRNA is essential in establishing a reading frame required for the SOS mechanism, latent splicing is suppressed when CAD-WT is complemented by ini-tRNA with a complementary anticodon (CAU). It is expected that abrogating SOS in an AUG to ACG mutant (CAD-Mut31), elicits latent splicing, which is rescued by a mutant in-tRNA, which carries a complementary anticodon (CGU) mutation, resulting in a reduced level of latent splicing (CAD-Mut31). These assumptions were verified [40]. Notably, recovery of SOS by ini-tRNA complementation is disrupted by NCL knockdown using siRNA. Scheme: gray box, exon; line, intron; blue box, latent exon; +(w) indicates weak latent splicing. (**b**,**c**) Experimental verification using the CAD minigene. HEK 293 cells were co-transfected with CAD-Mut31 (CAD31), which carries the mutated ACG start-codon; with CAD31 together with mutant ini-tRNA, in which the antisense codon was mutated to CGU (ini-CGU), as indicated. Co-transfection of CAD-Mut31 with mutant ini-tRNA and with si-RNA directed against NCL (NCLsi), disrupt the complementation. (**c**) Graphs represent an average of three independent experiments. The densitometric ratio of CAD31 was normalized to 100%. Adapted from ref. [46].

In an attempt to understand the rules of SOS, we showed that the presence of an in-frame stop codon plays a role in suppressing splicing from the latent sites. Specifically, we demonstrated, through a large series of mutations of gene constructs, that removal of stop codons led to activation of splicing at the latent SSs (latent splicing). These mutations included mutations in the stop codons to produce sense codons, and frame shift mutations by insertion or deletion of nucleotides upstream of the stop codons [35,36,39]. Three different lines of experiments ruled out the possibility that the mutations we made affected splicing through the damage of a splicing control element [10,45]: (i) by demonstrating that removal of only one of the two in-frame stop codons in a CAD-WT minigene construct (Figure 2a) was not enough to produce latent splicing; (ii) by revealing that mutating an in-frame stop codon to the remaining two stop codon sequences did not elicit latent splicing, but mutating the stop codon to a missense codon did; and (iii) by confirming that frame shift mutations, further away from the in-frame stop codon, elicited latent splicing [39].

Splicing control by SOS requires a starting point for the recognition of the mRNA reading frame, and the start codon AUG sequence was a likely candidate. Indeed, using mutagenesis, we demonstrated that AUG sequences are essential for SOS. Although protein translation does not seem to be required for SOS, the first AUG was shown to be necessary but not sufficient. The abrogation of SOS was attributed to a mutation in the AUG sequence, rather than interference with splicing control elements, because mutating nucleotides in the vicinity of the AUG sequence did not elicit latent splicing [12].

### 5.2. A Role for Initiator-tRNA in SOS

Our finding that mutations in the translation initiation codon, AUG, elicited latent splicing, even though the stop codons remained intact [12], supported the requirements for the conservation of the ORF for the SOS mechanism, with the AUG translation initiation codon as its starting point. This raised the possibility of the initiator-tRNA (ini-tRNA) as a potential SOS factor, through its recognition of the AUG. This was verified by demonstrating that the ini-tRNA has a regulatory role in pre-mRNA splicing, which is not connected with its function in protein translation. We demonstrated that abrogation of SOS, which occurred upon mutating the AUG translation initiation codon (Figure 2a), can be counterbalanced by ini-tRNA having complementary anticodon mutations to the AUG mutations, thus rescuing SOS. This rescue cannot be achieved by mutated elongator-tRNA [40]. Importantly, the ini-tRNA species that is proposed to participate in SOS resides in the cell nucleus and appears not to be charged with an amino acid. These findings indicate that the nuclear base-pairing of the uncharged ini-tRNA anticodon triplet with the initiation AUG sequence plays a key role in controlling the quality of splicing by the yet undeciphered SOS mechanism [40].

### 5.3. A Novel Role for Nucleolin (NCL) in SOS

In our efforts to decipher the SOS mechanism, we searched for partners of ini-tRNA in the nucleus. Starting with UV crosslinking followed by mass spectrometry analysis, we identified NCL as a protein that directly and specifically binds to ini-tRNA in the nucleus but not in the cytoplasm. NCL is an abundant RNA binding protein, with multiple cellular functions (e.g., synthesis and maturation of ribosomes in the nucleolus, a role in Pol II transcription, DNA repair, chromatin decondensation, and genome stability) which are not yet fully understood. Furthermore, NCL is known to play a role in cancer, in which its overexpression affects cell survival, proliferation, and invasion [47,48]. To establish the relevance of this association to SOS, we showed that ini-tRNA and NCL associated together with pre-mRNA. We further showed that SOS is NCL-dependent by using a construct mutated in the AUG, thereby abrogating SOS, in combination with a mutant ini-tRNA carrying an anti-codon mutation, which complements the AUG mutations and rescues SOS (Figure 2). Using this system, we showed that the recovery of SOS by ini-tRNA complementation is NCL-dependent, as the complementation is abrogated by knockdown of NCL (Figure 2b,c). Finally, NCL knockdown resulted in activation of latent splicing in hundreds of coding transcripts that have important cellular functions. We thus proposed NCL as the first protein component in a nuclear quality control mechanism that regulates splice site selection, thereby protecting cells from latent splicing that can generate defective mRNAs [46].

## 6. An Updated Model of the SOS Mechanism

**Figure 3:**
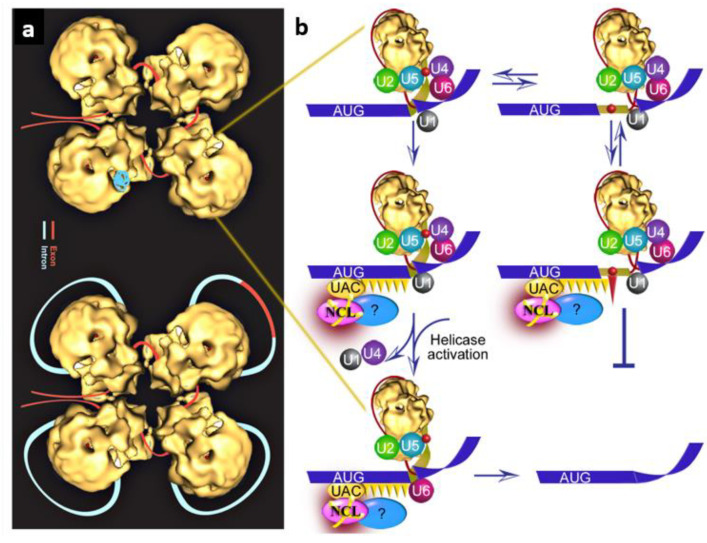
An updated speculative schematic model for the quality control function of SOS. (**a**) The supraspliceosome model [12,30]. Exon, red; intron, light blue. (Top) The folded pre-mRNA that is not being processed is protected within the cavities of the native spliceosome. (Bottom) When a staining protocol was used that allows visualization of nucleic acids, RNA strands and loops were seen emanating from the supraspliceosomes [12,30]. The RNA that was maintained in the cavity likely unfolded and looped-out under these staining conditions. In the looped-out scheme, an alternative exon is depicted in the upper right corner. (**b**) Zoom into one spliceosome. Left panel, splicing at the authentic 5′SS; right panel, splicing at the LSS. Blue stripes, exons; red line, intron; yellow narrow stripe, latent exon; red circle, in-frame stop codon; circles, U snRNPs; orange ellipse (UAC), initiator-tRNA; purple ellipse, NCL directly bound to ini-tRNA; blue ellipse, additional associated components; orange triangles, hypothesized triplet-binding proteins; red triangle, stop-codon-binding protein. Updated and adapted from refs. [40,46].

We previously proposed a speculative model for SOS as a sense triplet-recognition mechanism that can be interrupted by stop codon-binding proteins [40]. Our recent identification of NCL as a novel SOS factor enabled us to update our working model as a quality control mechanism within the endogenous spliceosome that acts prior to splicing (Figure 3). According to this model, initially, splice site combinations are selected through the combinatorial interplay of positive and negative regulatory signals present in the pre-mRNA, which are recognized by trans-acting factors. Next, approval or rejection of the splice choice is determined by the SOS mechanism. The first element comprises the recognition of the AUG sequence by the complementary anticodon (UAC) of the ini-tRNA, which is assembled in a complex with auxiliary proteins [40]. We propose that, at this stage, NCL can now be added in the model as a protein directly binding ini-tRNA, likely along with additional proteins. This step helps establish a register for the recognition of the reading frame [46]. The second SOS element involves the cooperative polymerization of protein(s) that bind triplets of nucleotides and in the absence of a PTC it reaches the selected 5′SS; this step is the quality control “confirmation” step of SS selection that triggers the remodeling of the spliceosome to its functional state (Figure 3b, left). The final element is suppression of splicing in the presence of a PTC, perhaps through a competing interaction with a stop-codon-binding protein (e.g., a release factor-like protein). The unproductive complex may undergo a conformational change and revert to a productive splicing complex involving the authentic 5′SS, as indicated by the double arrows in Figure 3b (right panel). In cases where the quality control mechanism fails, for example in instances of stress or cancer, downstream mechanism/s (e.g., NMD in the cytoplasm) may be engaged to safeguard the robust control of the system. Future work to identify additional components of the SOS mechanism, their interactions with the pre-mRNA and amongst them, and how these interactions affect latent splicing will help in deciphering the SOS mechanism.

## 7. Activation of LSS in Stress and Cancer

### 7.1. Activation of Latent Splicing under Stress

Analysis of the impact of environmental stress on gene expression revealed changes in the expression levels of mRNA of distinct sets of genes [49,50]. As SS choice is controlled by multiple biological factors, it is tempting to speculate that cellular stress could also affect SS selection of numerous mRNAs. In previous work, we have shown that heat shock elicited latent splicing in the CAD gene in Syrian hamster cells [31]. Furthermore, heat shock also activated latent splicing in tested *C. elegans* transcripts [36]. We further showed that exposing Syrian hamster cells to γ irradiation, hypoxia, cold shock, and heat shock elicited latent splicing in endogenous CAD mRNA, with heat shock causing the strongest effect [31]. We therefore examined the global effect of heat shock on latent splicing using a splicing-sensitive microarray, revealing activation of splicing in 508 latent sites. It should be pointed out that this number of activated LSSs is a lower limit, because the latent transcript, which contains PTCs, is downregulated by NMD in the cytoplasm [31].

### 7.2. Activation of Latent Splicing in Cancer

Female breast cancer is the second most commonly diagnosed cancer and the fifth leading cause of cancer-related deaths worldwide [51,52,53,54,55]. Analyzing data mined from the Gene Expression Omnibus (GEO) of MCF-7 breast cancer (BC) cells as compared to MCF-10A non-malignant breast cells [56] revealed activation of latent splicing in 794 latent sites [31]. Brain cancers are characterized by high morbidity and mortality, owing to their localization and often local invasive growth [57,58]. Gliomas are the most common primary central nervous system tumors in adults, and despite advances in treatments, the prognosis for most glioma patients remains poor [59]. We performed similar analyses of activation of latent splicing in a number of gliomas, using data available in the GEO database [60]. This analysis revealed that in glioblastoma tumors, 409 latent sites were activated, while in oligodendroglioma (OD) samples the number of activated LSSs were 853 in grade II and 612 in grade III [31]. Analysis of overlapping genes expressing latent mRNAs in the three gliomas revealed a highly significant group of 178 LSS that showed activation of latent splicing in all three glial tumors [31].

**Figure 4:**
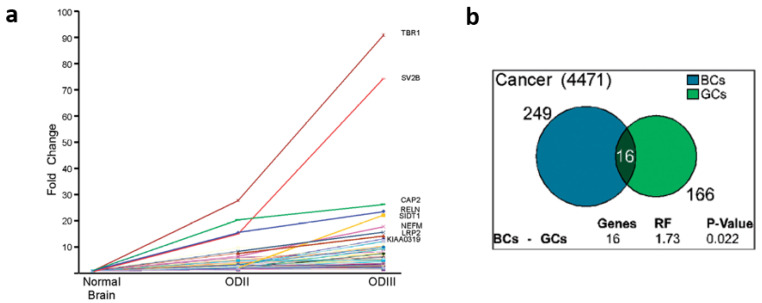
Activation of LSS in cancer: (**a**) Correlation between the level of activation of latent splicing and the severity of OD. The graph depicts fold changes in the level of latent splicing in 125 gene transcripts whose latent splicing expression increased from normal cells to ODII and further increased in ODIII. Names of the top-scoring gene transcripts are indicated. (**b**) Overlap between activated LSSs in breast cancer (BC) MCF-7 cells, and in glial tumors (GC, glioma cancer). Adapted from ref. [31].

Next, we searched for a correlation between activation of latent splicing and increased malignancy by looking for genes in which the extent of latent splicing activation increased between samples of Oligodendroglioma Grades II (ODII) and III (ODIII), as they exemplify different stages of severity of the same malignant tumor. The analysis revealed 125 mRNAs for which the extent of latent splicing activation was higher in the more aggressive ODIII than in ODII or normal cells (Figure 4a). The two transcripts with the most latent splicing activation are as follows: (i) the transcript of T box brain 1 transcription factor (TBR1), which has a T-box-DNA binding motif and encodes for a transcription factor implicated in regulating developmental processes; and (ii) the transcript of synaptic vesicle glycoprotein 2B (SV2B), proposed to be involved in the regulation of secretion in neural and endocrine cells. In both cases, we found significant activation of latent splicing in ODIII compared to ODII and normal cells (Figure 4a) [31].

**Figure 5:**
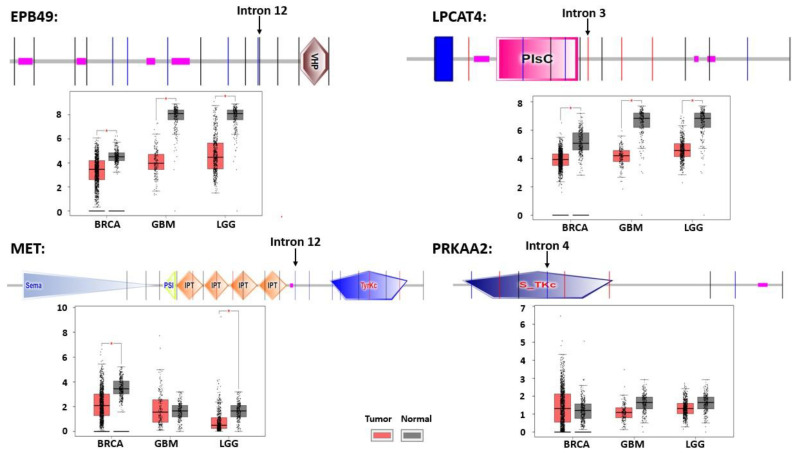
Representative gene transcripts which showed activation of latent splicing in both breast cancer cells and neuronal tumors (MCF-7 breast cancer cells; glioma and oligodendroglioma grade II and III tumors) [31]. Diagram of the protein domains, based on SMRT EMBL database, where the location of the activated LSS [31] is marked; the vertical lines refer to intron locations. EPB49, contains a low complexity region (pink box) and a VHP (Villin headpiece) catalytic domain; LPCAT4, harbors a transmembrane domain (blue box), a low complexity region (pink box), and a PlsC (Phosphate acyltransferase) domain; MET, contains a Sema (semaphorin domain), PSI domain (found in several different extracellular receptors, including plexins, which are involved in the development of neural and epithelial tissues), IPT (Ig-like, plexins, transcription factors) domain, low complexity region (pink box), and the catalytic domain TyrKc (Tyrosine kinase); PRKAA2, carries a catalytic domain S_TKc (Serine/Threonine protein kinases), and a low complexity region. Below each protein diagram, the expression of the respective gene is presented, as tested in 1085, 163, and 518 BRCA, GBM and LGG tissue samples, respectively, and in 291, 207, and 207 non-tumor tissues, based on data from GEPIA. BRCA, breast invasive carcinoma; GBM, glioblastoma multiforme; LGG, brain lower grade glioma. Red star indicates significant difference between tumors and normal tissues (*p* value ≤ 0.01).

We then looked for cases of activation of latent splicing which occur in both the gliomas and the BC cells, by comparing the cases of activation of latent splicing in MCF-7 BC cells with those of the three glial tumors, taking into account only genes that are expressed in both BC cells and glial tumors (249 out of 260 LSSs in MCF-7 cells; 166 out of 178 LSSs in the glial tumors). We identified 16 gene transcripts in which latent splicing is activated in both MCF-7 BC cells and glioma tumors (Figure 4b and Table 1) [31]. This list includes genes with important cellular functions such as kinases and tumor suppressors, as well as genes involved in the repair of double-strand breaks. The potential effect on cellular function is elaborated on four out of the sixteen proteins as follows (Figure 5):

(i) EPB49—Erythrocyte Membrane Protein Band 4.9, also known as DMTN (Dematin Actin Binding Protein). EPB49 is a membrane-cytoskeleton-associated protein with F-actin-binding activity that induces F-actin bundle formation and stabilization. It plays an important role in maintaining cell morphological integrity and in regulating cell migration. It also acts as a tumor suppressor, inhibiting malignant cell transformation [62]. EPB49 methylation changes can serve as a biomarker for BC progression [63]. The catalytic domain of this gene, the VHP-F-actin bundling domain, is located in the carboxy terminus. We found activation of an LSS in intron 12, introducing a PTC in MCF-7 cells, glioblastoma, and OD. Since the catalytic domain of this gene is located downstream to the activated LSS, the introduction of a PTC likely leads to the loss of this protein’s activity. This is in correlation with the hypermethylation of EPB49, seen in BC cells [63]. (ii) LPCAT4—Lysophosphatidylcholine Acyltransferase 4. This protein is involved in biosynthesis of glycerolipids and is known to play an important role in phospholipid remodeling in the brain [64]. Its catalytic domain, phosphate acyltransferases (PlsC), plays a role in phospholipid biosynthesis and is located within exons 2–5. Because we found activation of an LSS within intron 3 of this gene transcript in both BC and GC [31], leading to interruption in the catalytic domain, the activation of this LSS likely impairs protein activity. (iii) MET—Proto-Oncogene, Receptor Tyrosine Kinase, Hepatocyte/Growth Factor Receptor. This receptor tyrosine kinase transduces signals from the extracellular matrix into the cytoplasm by binding to hepatocyte growth factor. It regulates many physiological processes including proliferation, scattering, morphogenesis, and survival in response to environmental stimulation [65]. We found activation of an LSS in intron 12 introducing a PTC in BC and GC [31], which likely results in the expression of a protein that lacks the TyrKc catalytic domain. (iv) PRKAA2—Protein Kinase, AMP-activated, Alpha 2 Catalytic Subunit. This AMP-activated protein kinase (AMPK) is a critical sensor of cellular energy and nutrient levels, and loss of AMPK or deregulation of its activity has been linked to cancer [66,67]. We found activation of an LSS in intron 4 [31], introducing a PTC that disrupts the catalytic domain in BC and GC, presumably resulting in downregulation of its activity.

Figure 5 shows data of the downregulated expression found for these four genes in BC, GBM, and LGG as compared to the relevant normal tissues from GEPIA (Gene Expression Profiling Interactive Analysis) based on RNA sequence data [68]. With respect to the expression of MET in MCF-7 cells, it should be noted that, although cases of increased MET expression in MCF-7 cells were reported [69,70], the data from GEO (GSE19154) [56], which we analyzed for latent splicing activation, showed decreased expression of MET in MCF-7 compared to MCF-10 cells. Notably, this downregulation in expression is in agreement with the expected downregulation of protein function due to latent splicing activation in all four discussed proteins in BC MCF-7 cells as well as glioblastoma and OD tumors, highlighting the relevance of SOS to human health and cancer.

In addition to the four examples we elaborated upon, in which latent splicing activation overlaps between BC cells and gliomas, resulting in aberrant AS and likely leading to the production of damaged proteins that lack an active domain, there are an additional twelve common gene transcripts affected in both types of cancers [31]. We also identified thousands of LSS that were activated in MCF-7 BC cells, glioblastoma, and OD grades II and III. It should be noted that these numbers are only a lower limit, due to the filters we used in the bioinformatics analysis and the limitations of the array [31]. An additional important reason that the actual number of cancer-activated LSSs might be higher than described [31], is because activation of latent splicing results in the generation of mRNA that has a PTC, and thus it is targeted by the NMD mechanism in the cytoplasm [31]. While it is yet too early to estimate the impact of latent splicing on cancer, these findings indicate a possible linkage between oncogenesis and the elevation in expression of a series of LSSs. It is anticipated that further investigation into activation of latent splicing in cancer could lead to the identification of novel diagnostic approaches and therapeutic targets. It is also possible that upon latent splicing activation in stress and in cancer, novel polypeptides encoded by the latent exons might be identified as external and dangerous signals by the immune system of the host. Therefore, we focus our attention on the thousands of latent SSs that are activated in cancer and encode damaged proteins, having potentially harmful effects on cell metabolism, as unexplored candidate novel targets for cancer diagnostics and treatment.

## 8. Conclusions

The SOS quality control mechanism protects cells from activation of the numerous LSSs present in introns, whose activation could generate transcripts with PTCs that might be toxic to cells. Notably, under stress and in cancer, SOS is abrogated, resulting in the expression of thousands of unexplored aberrant mRNAs. These mRNAs are from broad functional groups, including mRNAs implicated in cell differentiation and proliferation. Therefore, we believe that elucidating the SOS mechanism is relevant to cancer. A specific example of this connection is the case of OD, in which a correlation was found between the level of activation of latent splicing and the severity of the disease. An increase in the expression levels of latent splicing transcripts from normal cells to ODII and a further increase in ODIII was found for 125 gene transcripts, portraying novel markers for OD. Another important example is the case of 16 gene transcripts, which reveals activation of latent splicing in both BC cells and glioma tumors. Latent splicing activation in these 16 gene transcripts represents novel targets in the fight against cancer. Furthermore, we propose examples of how latent splicing might disrupt the expression and function of translated proteins encoded by the latent mRNAs in four out of sixteen gene transcripts. Importantly, expected changes in expression levels in cancer are in good correlation with data of changes in expression levels from tumors compared to non-malignant samples. We therefore conclude that targeting genes that show activation of latent splicing in cancer might lead to novel avenues in cancer diagnostics and treatment.

## Figures and Tables

**Table 1:** Gene transcripts with activated LSS in breast cancer cells and gliomas.

Gene Symbol	Gene Name	LSS Location	LSS Position ^a^	IVS No.	No. PTCs in Latent Exon ^b^	LSS Score ^c^	Authentic Score ^c^
ATP5F1	ATP synthase, H+ transporting, mitochondrial Fo complex, subunit B1	chr1:111997965	988	3	21	89.77	96.83
CALY	Calcyon neuron-specific vesicular protein	chr10:135140854	554	3	3	94.43	84.55
CLMP	CXADR-like membrane protein	chr11:122945289	120	6	6	90.58	81.58
DHCR24	24-dehydrocholesterol reductase	chr1:55330677	298	6	3	91.43	97.36
EFHD2	EF-hand domain family, member D2	chr1:15754589	810	3	9	90.54	96.22
EPB49	Erythrocyte membrane protein band 4.9 (Dematin)	chr8:21938253	284	12	3	91.81	42.29
EPHX4	Epoxide hydrolase 4	chr1:92509409	873	3	22	91.46	90.88
EXOSC1	Exosome component 1	chr10:99197087	369	6	7	91.5	77.12
LPCAT4	Lysophosphatidylcholine acyltransferase 4	chr15:34656702	506	3	5	90.39	94.18
MET	Met proto-oncogene (hepatocyte growth factor receptor)	chr7:116410706	862	12	15	90.68	89.46
PRKAA2	Protein kinase, AMP-activated, alpha 2 catalytic subunit	chr1:57159011	837	4	29	90.46	85.41
PRPF4B	Serine/threonine-protein kinase PRP4 homolog	chr6:4059800	753	14	14	92.37	94.63
RAD21	Double-strand-break repair protein rad21 homolog	chr8:117878362	462	2	13	89.45	92.94
RDH12	Retinol dehydrogenase 12	chr14:68193242	371	6	6	90.8	91.36
SLC25A12	Solute Carrier Family 25 (Mitochondrial Carrier, Aralar), Member 12	chr2:172670735	895	10	10	90.43	96.95
WDR66	Cilia- and flagella-associated protein 251	chr12:122400536	469	15	4	89.19	97.7

^a^ Number of nt downstream from the authentic 5′SS. ^b^ Number of in-frame stop codons within the latent exon. ^c^ Scores according to ref [61]. Table composed from data of Ref. [31].

## Abbreviations

SOS	Suppression of Splicing
NMD	Nonsense Mediated mRNA Decay
Pol II	RNA Polymerase II
RNP	Ribonucleoprotein
snRNP	small nuclear RNP
AS	Alternative Splicing
SS	Splice Site
hnRNP	heterogenous nuclear Ribonucleoproteins
PTCs	Premature Termination Codons
NAS	Nonsense-mediated Altered Splicing
ORF	Open Reading Frame
LSS	Latent 5′ Splice Site
Ini-tRNA	initiator-tRNA
NCL	Nucleolin
CAD31	CAD-Mut31
GEO	Gene Expression Omnibus
TBR1	T box brain 1
SV2B	Synaptic Vesicle glycoprotein 2B
OD	Oligodendroglioma
ODII	Oligodendroglioma Grade II
ODIII	Oligodendroglioma Grade III
BC	Breast Cancer
GC	Glioma Cancer
EPB49	Erythrocyte membrane protein band 4.9 (Dematin)
LPCAT4	Lysophosphatidylcholine acyltransferase 4
MET	Met proto-oncogene (hepatocyte growth factor receptor)
PRKAA2	Protein kinase, AMP-activated, alpha 2 catalytic subunit
VHP	Villin Headpiece
PlsC	Phosphate acyltransferases
Sema	Semaphorin domain
PSI	Plexins, Semaphorins and Integrins
IPT	Ig-like, plexins, transcription factors
TyrKc	Tyrosine kinase
S_TKc	Serine/Threonine protein kinases
BRCA	Breast invasive carcinoma
GBM	Glioblastoma multiforme
LGG	Brain Lower Grade Glioma
GEPIA	Gene Expression Profiling Interactive Analysis
DMTN	Dematin
AMPK	AMP-activated protein Kinase
